# Proteomics of blood extracellular vesicles in inflammatory respiratory diseases for biomarker discovery and new insights into pathophysiology

**DOI:** 10.1186/s41232-024-00351-4

**Published:** 2024-09-18

**Authors:** Takahiro Kawasaki, Yoshito Takeda, Atsushi Kumanogoh

**Affiliations:** 1 Department of Respiratory Medicine and Clinical Immunology, Osaka University Graduate School of Medicine, Suita, Osaka Japan; 2 Department of Immunopathology, World Premier International Research Center Initiative (WPI), Immunology Frontier Research Center (IFReC), Osaka University, Suita, Osaka Japan; 3 Center for Infectious Diseases for Education and Research (CiDER), Osaka University, Suita, Osaka Japan; 4 Integrated Frontier Research for Medical Science Division, Institute for Open and Transdisciplinary Research Initiatives (OTRI), Osaka University, Suita, Osaka Japan; 5 Japan Agency for Medical Research and Development – Core Research for Evolutional Science and Technology (AMED–CREST), Osaka University, Suita, Osaka Japan; 6 Center for Advanced Modalities and DDS (CAMaD), Osaka University, Osaka, Japan

**Keywords:** Interstitial lung disease, Bronchial asthma, Chronic obstructive pulmonary disease, Respiratory infections, Blood extracellular vesicles, Proteomics

## Abstract

**Background:**

Inflammatory respiratory diseases, such as interstitial lung disease (ILD), bronchial asthma (BA), chronic obstructive pulmonary disease (COPD), and respiratory infections, remain significant global health concerns owing to their chronic and severe nature. Emerging as a valuable resource, blood extracellular vesicles (EVs) offer insights into disease pathophysiology and biomarker discovery in these conditions.

**Main body:**

This review explores the advancements in blood EV proteomics for inflammatory respiratory diseases, highlighting their potential as non-invasive diagnostic and prognostic tools. Blood EVs offer advantages over traditional serum or plasma samples. Proteomic analyses of blood EVs have revealed numerous biomarkers that can be used to stratify patients, predict disease progression, and identify candidate therapeutic targets. Blood EV proteomics has identified proteins associated with progressive fibrosis in ILD, offering new avenues of treatment. In BA, eosinophil-derived EVs harbor biomarkers crucial for managing eosinophilic inflammation. Research on COPD has also identified proteins that correlate with lung function. Moreover, EVs play a critical role in respiratory infections such as COVID-19, and disease-associated proteins are encapsulated. Thus, proteomic studies have identified key molecules involved in disease severity and immune responses, underscoring their role in monitoring and guiding therapy.

**Conclusion:**

This review highlights the potential of blood EV proteomics as a non-invasive diagnostic and prognostic tool for inflammatory respiratory diseases, providing a promising avenue for improved patient management and therapeutic development.

## Background

Inflammatory respiratory diseases, including interstitial lung disease (ILD), bronchial asthma (BA), chronic obstructive pulmonary disease (COPD), and other infectious diseases, are significant global health concerns. Although nonmalignant, these conditions are major causes of morbidity and mortality worldwide and are characterized by chronic inflammation, varying degrees of airway obstruction, and tissue remodeling, which can lead to severe respiratory failure. Despite their clinical importance, no effective biomarkers have been identified that reflect the pathophysiology of these diseases or can be used in personalized medicine, although detailed mechanisms have been elucidated.

Extracellular vesicles (EVs) are lipid bilayer-enclosed vesicles secreted by all types of cells, and they cannot replicate on their own [1]. They are known to play crucial roles as intercellular communication agents in pathophysiological processes. EVs contain lipids, proteins, and nucleic acids. Based on differences in size and production mechanisms, there are various classifications of EVs. Exosomes are vesicles derived from endosomal membranes and are released through multivesicular body (MVB) [1]. In contrast, ectosomes are vesicles the directly bud from the plasma membrane. With respect to the size of EVs, small EVs (50–150 nm in diameter) including exosomes are the most abundant in the body fluids, whereas large EVs (≥ 1 μm in diameter) such as apoptotic bodies are present in smaller numbers [2]. These vesicles are released into various body fluids, including blood, urine, saliva, and breast milk, and are taken up by target cells through direct membrane fusion, ligand-receptor interactions, or endocytosis [3]. Most of circulating EVs are thought to be cleared by uptake in target organs [3].

Recent advancements in EV research have opened new opportunities for elucidating the pathogenesis of various diseases, including malignancies and inflammatory conditions, and for discovering novel biomarkers. The regulation of EV biosynthesis and cargo selection is crucial for identifying pathology-specific profiles essential for clinical applications [4, 5]. Among the various omics approaches, proteomics is directly related to phenotypes, and proteins have been most frequently used as biomarkers in clinical practice [6]. Particularly in cancer research, proteins of blood EVs, which include plasma or serum EVs, are being actively explored as promising tools for liquid biopsy, offering non-invasive methods for diagnosis and prognostic evaluation [7–9].

**Fig. 1:**
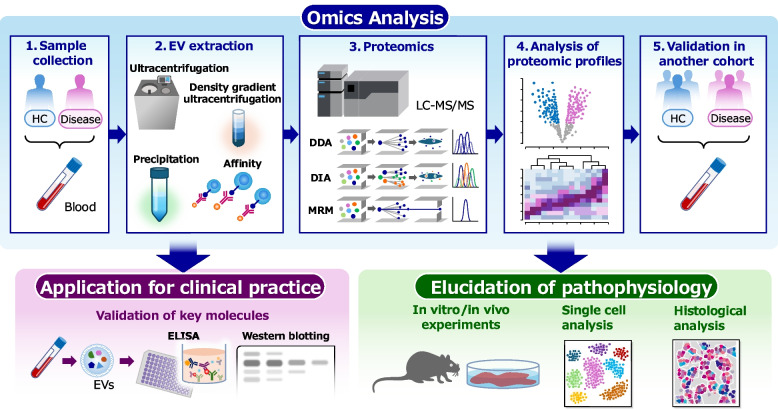
Workflow for proteomics studies in inflammatory lung diseases using blood EVs. First, from blood samples collected from patients and control cases, EVs are extracted. Proteomics, mainly mass spectrometry-based proteomics, is conducted on these EV samples. The obtained proteomic data are compared between patients and controls to identify candidate biomarkers or key molecules associated with the diseases. The results are validated in an independent cohort to confirm the promising candidates. Subsequent studies include the validation of these candidates using clinically applicable measurement systems and biological analyses to elucidate the pathophysiological mechanisms regulated by the identified molecules. Abbreviations: LC–MS/MS, liquid chromatography with tandem mass spectrometry; DIA, data independent acquisition; DDA, data dependent acquisition; MRM, multiple reaction monitoring

Lungs are the largest organ in the body with extensive blood circulation; therefore, blood EV proteomics is a valuable approach for biomarker discovery and understanding the pathogenesis of inflammatory respiratory diseases. This review details the technical advancements and significant findings in blood EV proteomics research within the context of inflammatory respiratory diseases and discusses their potential clinical applications and prospects (Fig. 1).

### Proteomics technology for EVs

Recently, blood EVs have attracted considerable attention as valuable resources for biomarkers, and they can be easily and repeatedly collected from blood and, unlike plasma or serum, are protected from degradation by proteases, retaining disease-related molecular information [1, 10]. Furthermore, EVs lack abundant proteins such as albumin and globulins, making them more suitable for deep proteomic analysis than plasma or serum [11].

Advances in proteomic techniques have been remarkable in recent years. Mass spectrometry (MS) techniques are primarily used in high-depth proteomics. Typically, mixtures of peptide fragments are introduced into an analytical system, liquid chromatography/mass spectrometry (LC–MS/MS), in which a tandem mass spectrometer is connected to a low-flow liquid chromatograph [12]. Conventional peptide measurements rely on the technique of data-dependent acquisition (DDA), which automatically selects ionized peptides for fragmentation based on the detection intensity of each ion [13]. This method can be used to identify numerous proteins.

Recently, however, a new MS mode, data-independent acquisition (DIA), has been developed. In this method, ions within a set m/z range are simultaneously subjected to fragmentation while shifting the m/z window rather than being selected on an ionized peptide-by-peptide basis. This revolutionary method, also known as next-generation proteomics, enables comprehensive protein identification at greater depths with higher precision than DDA [14, 15].

By utilizing blood EVs and analyzing them using state-of-the-art proteomic techniques, it has become possible to measure a wide range of pathophysiology-related proteins, even in trace amounts. Consequently, biomarker research for various diseases, particularly cancer, has advanced, and a new understanding of their pathogenesis has been achieved. This strategy is also being applied in research on inflammatory respiratory diseases.

### Blood EV proteomics in ILDs

Interstitial lung diseases (ILDs) comprise a category of disorders marked by inflammation and fibrosis of the lung interstitium, leading to reduced pulmonary function. ILDs include various underlying conditions, such as idiopathic pulmonary fibrosis (IPF), connective tissue disease-related ILD, and chronic hypersensitivity pneumonitis. Increasing attention is being given to a subset of these diseases that progress to a phenotype characterized by extensive fibrosis, known as progressive fibrosing ILD (PF-ILD) [16] or progressive pulmonary fibrosis (PPF) [17]. It is estimated that 30–40% of ILD cases develop progressive fibrosis with a poor prognosis, exhibiting a median survival of 2.5–3.5 years [18].

The role of EVs in fibrosis pathogenesis has attracted considerable attention. For example, WNT5A-positive EVs, produced predominantly by fibroblasts, promote fibrosis [19]. Aging fibroblasts induce fibroblast infiltration via fibronectin in EVs [20]. Proteomic analyses of fibroblast-derived EVs from patients with IPF have revealed a higher number of proteins involved in fibrogenic processes than those from healthy individuals [21]. While EVs from alveolar epithelial cells have been implicated in fibroblast activation [22], EVs from the airway epithelial cells of patients with IPF promote epithelial cell senescence and induce inflammation [23].

Therefore, blood EV proteomics holds promise for the identification of key disease molecules and biomarkers (Table 1). For example, Adduri et al. performed LC–MS/MS proteomic analysis on 163 plasma EV samples, including those from patients with IPF, CHP, and NSIP, and healthy controls, identifying EV proteins SFTPB, ALDOA, HMGB1, CALML5, and TLN1 as useful markers for distinguishing IPF from other ILDs [24]. In another study, serum EVs from patients with ILD exhibiting progressive fibrosis other than IPF were analyzed using DIA-based next-generation proteomics, revealing 2420 proteins. Among them, SFTPB is a biomarker for predicting progressive fibrosis, which was confirmed using immunohistochemical analyses of patient lung tissues. SFTPB, a pulmonary surfactant protein produced by alveolar epithelial cells, is normally shed and matures. The mature form, which is abundant in the serum, is not a reliable predictor of progressive fibrosis. However, the pro-form is secreted into EVs during fibrosis and protected from shedding, and can accurately predict progressive fibrosis [25]. Interestingly, in single-cell RNA sequencing of lung tissues of a murine model of pulmonary fibrosis, the expression of SFTPB was upregulated in alveolar epithelial cells before the fibrotic phase, suggesting that this molecule is associated with profibrotic pathophysiology. Furthermore, Bayesian network integration analysis of the serum EV proteome with clinical data revealed IPF-specific network including modules related to TGF-β signaling and complement pathways, when comparing patients with IPF to healthy subjects [26].

**Table 1:** Blood EV proteomics for inflammatory respiratory diseases

References	Disease	Technology	MS mode	Feature	Biomarkers	Disease severity predicted by the biomarkers
Adduri, et al. [24]	ILD	LC–MS/MS	DDA	Diagnosis for IPF	SFTPB, ALDOA, HMGB1, CALML5, and TLN1	Diagnostic/classification use
Enomoto, et al. [25]	ILD	LC–MS/MS	DIA	Diagnosis for PPF	SFTPB	Unfavorable
Tomoto, et al. [26]	ILD	LC–MS/MS	DIA	Estimation of IPF‑specific protein network	-	-
Yoshimura, et al. [37]	BA	LC–MS/MS	DIA	Diagnosis for eosinophilic BA	Gal-10	Unfavorable
Koba, et al. [45]	COPD	LC–MS/MS	DDA	Diagnosis for COPD	Fibulin-3	Unfavorable
Jung, et al. [46]	COPD	EV array analysis		Diagnosis for acute exacerbation of COPD	CD45 and CD28	Diagnostic/classification use
Mao, et al. [60]	COVID-19	LC–MS/MS	DIA	Characteristics and residual traces of recovered COVID-19 patients	Proteins associated with coagulation activity, inflammatory reaction, immune response, and low organ function	Diagnostic/classification use
Krishnamachary, et al. [62]	COVID-19	PEA	-	Diagnosis of severe patients	TF, CD163, and EN-RAGE	Unfavorable
Fujita, et al. [61]	COVID-19	LC–MS/MS	DDA	Predictive biomarkers of severe patients	COPB2	Favorable
Lam, et al. [49]	COVID-19	LC–MS/MS	DDA	Proteomics change of different temporal stages of COVID-19	C1r, C1s	Diagnostic/classification use
Kawasaki, et al. [63]	COVID-19	LC–MS/MS	DIA	Predictive biomarkers of refractory patients	MACROH2A1	Unfavorable
Mehaffy, et al. [70]	M. tuberculosis	MRM-MS	-	Identification of peptides associated with LTBI	glutamine synthetase (GlnA1) enzyme	Diagnostic/classification use
Cheng, et al. [71]	Pediatric pneumonia	LC–MS/MS	DDA	Immune signatures of children with pneumonia	SERPINA1, ITIH4, IGLV6-57, HIST2H3A, HIST1H4, HIST1H2BL, COL1A1, ANXA2, COL2A1, ACAN, DSP, XP32, DSC1, HAPLN1, and DSG1	Diagnostic/classification use

*Abbreviations*: *ILD* interstitial lung disease, *IPF* idiopathic pulmonary fibrosis, *PPF* progressive pulmonary fibrosis, *BA* bronchial asthma, *COPD* chronic obstructive pulmonary disease, *LTBI* latent tuberculosis infection, *LC–MS/MS* liquid chromatography with tandem mass spectrometry, *MS* mass spectrometry, *PEA* proximity extension analysis, *DIA* data-independent acquisition, *DDA* data-dependent acquisition

Despite the potential of blood EV proteomics, the field remains in its infancy, and relatively fewer studies have focused on miRNAs [27]. This area of research holds substantial promise for further development, particularly when considering the heterogeneity of ILDs. Identifying molecules that facilitate patient stratification and control of common pathological processes, such as PPF, remains a critical issue.

### Blood EV proteomics in BA

BA is a common chronic allergic airway disease characterized by reversible airflow limitation in the peripheral airways, leading to clinical symptoms such as wheezing and dyspnea [28]. Approximately 300 million patients suffer from asthma worldwide [29], with asthma-related deaths reported to be approximately 200,000 per year [30], making it a major global health challenge. The pathogenesis of BA involves immune cells, such as eosinophils, Th2 lymphocytes, and type 2 innate lymphocytes, as well as non-immune cells including airway epithelial cells and smooth muscle cells.

Cytokine-mediated interactions, including those between TSLP, interleukin (IL)-13, IL-33, and IL-5, play crucial roles in promoting disease; these cytokines are targets for biologic agents [31, 32]. The significance of EVs in the pathogenesis of BA has been highlighted by numerous studies. For example, eosinophil-derived EVs are released in response to inflammatory stimuli [33], dendritic cell-derived EVs promote CD4^+^T cell proliferation and Th2 differentiation [34], and airway epithelial cell-derived EVs can induce airway inflammation [35]. Notably, Cañas et al. conducted proteomic analysis on EVs extracted from peripheral blood eosinophils of patients with asthma and healthy subjects and demonstrated that eosinophil-derived EVs from patients with asthma promote ROS production and eosinophil migration [36].

By focusing on the proteomics of serum EVs, Yoshimura et al. identified Galectin-10 as a biomarker of eosinophilic asthma using DIA MS [37]. This protein demonstrated superior diagnostic capability compared with peripheral blood eosinophil counts and correlated with clinical parameters, such as airflow obstruction and mucus plug. Moreover, Galectin-10 released by eosinophils during EETosis has been found to correlate with the degree of EETosis in BA lung tissue and nasal mucosal tissue in chronic rhinosinusitis with nasal polyps, suggesting that Galectin-10 in EVs may reflect the pathogenesis of eosinophilic inflammation in asthma (Table 1).

Although eosinophil-derived EVs, which are crucial for the development of BA, are a rich source of biomarkers and important disease molecules, it is essential to focus on EVs derived from cells other than eosinophils to identify biomarkers and elucidate the pathophysiology of non-eosinophilic asthma in the future. Compared with eosinophilic asthma, non-eosinophilic asthma currently lacks sufficient biomarkers and therapeutic options, and the proteomics of blood EVs may offer a valuable research tool to address this unmet need.

### Blood EV proteomics in COPD

COPD is a long-term lung condition marked by persistent airflow obstruction that primarily results from the prolonged inhalation of tobacco smoke. Inhalation of harmful substances induces inflammation, oxidative stress, and alveolar cell apoptosis, triggering alveolar destruction and remodeling [38]. One of the primary mechanisms by which EVs contribute to the pathogenesis of COPD involves the degradation of the extracellular matrix. Neutrophil elastase level in EVs derived from multinucleated cells is higher in the lungs of patients with COPD than in the lungs of healthy subjects, and the transfer of these EVs collected by bronchoalveolar lavage of patients with COPD to mice can induce COPD-like symptoms [39]. In addition, in vitro studies have shown that EVs released from airway epithelial cells in response to cigarette smoke exposure promote MMP-1 production via CCN1 (Cyr61) [40] and macrophages produce MMP14-rich EVs when stimulated with cigarette smoke extract [41]. EVs derived from macrophages after atmospheric fine particulate matter (PM) stimulation induce IL-6 and TNFα production in lung epithelial cells [42]. Similarly, mononuclear cells stimulated by cigarette smoke extract produce microparticles that induce proinflammatory mediators such as ICAM-1 and IL-8 in lung epithelial cells [43]. Moreover, EVs from airway epithelial cells stimulated with cigarette smoke extract have been shown to induce M1 macrophage polarization [44]. These findings suggest that EVs play a role in promoting chronic airway inflammation.

Several proteomic studies have been conducted on blood EVs in patients with COPD (Table 1). For example, Koba et al. performed a proteomic analysis of serum EVs from patients and mouse models with COPD to identify common biomarkers [45]. The authors identified Fibulin-3 as an elevated protein in patients with COPD, correlating with the extent of emphysema on CT scans and reduced pulmonary function. Notably, Fibulin-3-knockout mice spontaneously developed emphysema, suggesting that key pathological molecules were encapsulated within EVs. Distinguishing acute exacerbations of COPD (AECOPD) from bacterial pneumonia, often presenting as acute respiratory failure, is clinically challenging. Jung et al. conducted a comprehensive examination of the surface proteins on plasma EVs from patients with community-acquired pneumonia and AECOPD using protein microarrays and successfully identified CD45 and CD28 as the most useful markers for differentiation [46].

To date, blood EV omics analysis in COPD has mainly focused on miRNAs [47, 48]; however, as discussed above, substantial evidence suggests that EV proteins play a critical role in the pathogenesis of COPD. Given the lack of curative treatments for COPD, identifying molecules through blood EV proteomics that influence disease pathogenesis or serve as therapeutic targets is crucial.

### Blood EV proteomics in respiratory infections

The role of EVs in the pathogenesis of respiratory infections, including viral and bacterial infections, is being increasingly recognized. In particular, the COVID-19 pandemic, which has led to numerous severe cases and deaths, has promoted extensive research on blood EV proteomics and EV involvement in disease pathogenesis.

#### EVs in viral infections

The involvement of EVs in COVID-19 pathogenesis has been extensively investigated. EVs derived from SARS-CoV-2-infected cells contribute to disease propagation by transporting viral particles and inflammatory molecules [49–51]. EVs from epithelial cells containing SP-C can induce chronic inflammation in the heart [52]. Moreover, endothelial cell-derived serum EVs are correlated with hospitalization mortality [53]. In immune cells, EVs from dendritic cells stimulated by viral proteins activate CD4^+^ and CD8^+^ T-cells [54], and neutrophil elastase in EVs has been reported to cause endothelial cell damage [55].

EVs are involved in the pathogenesis of other viral infections by inducing inflammation. For instance, in RS virus infection, EVs from infected epithelial cells show altered protein profiles, leading to elevated levels of chemokines including CCL20 [56]. EVs derived from infected lung epithelial cells stimulate monocytes and epithelial cells to secrete proinflammatory mediators [57]. Previous studies have suggested that EVs may have protective functions against infections. In influenza infection, EVs have a role in host defense including neutralizing the virus [58]. Additionally, the uptake of macrophage-derived EVs by alveolar epithelial cells increases endosomal acidification and inhibits viral nuclear transfer and replication [59].

Most studies on blood EV proteomics in viral infections have focused on COVID-19, revealing critical insights through various proteomic methods (Table 1). Mao et al. used MS in the DIA mode to analyze plasma EVs from patients with COVID-19 in the recovery phase, identifying a total of 394 proteins, with 174 differentially expressed proteins associated with coagulation, inflammation, immune response, and organ dysfunction [60]. Similarly, Fujita et al. employed DDA mode proteomics on serum EVs from patients with COVID-19 and discovered that the protein COPB2 could diagnose severe illness post-admission with high accuracy (AUC = 1.0 in the discovery set, AUC = 0.85 in the validation set) [61]. These findings underscore the utility of the DIA and DDA methods in identifying critical proteins involved in COVID-19 pathogenesis. In addition to these methods, Krishnamachary et al. used the proximity extension assay (PEA) on plasma EVs from 84 hospitalized patients with COVID-19 to uncover enriched pathways related to coagulation and inflammation. The authors identified EN-RAGE, TF, and IL-18R1 as proteins that strongly correlated with disease severity and length of hospitalization, suggesting that these proteins are key markers in COVID-19 pathology. Furthermore, EVs from patients with COVID-19 have been shown to induce the apoptosis of pulmonary microvascular endothelial cells in relation to disease severity, highlighting the potential of PEA in identifying biomarkers [62]. Multiomics analyses provided significant insights. Lam et al. combined DDA proteomics with lipidomics to analyze plasma EVs from patients with COVID-19 at different stages of the disease. The authors revealed enrichment of pathways involved in the complement system, coagulation cascade, and platelet activation, with decreased levels of C1r and C1s during the symptomatic phase compared to the asymptomatic phase, indicating potential biomarkers for disease progression [49]. Kawasaki et al. used DIA mode proteomics on serum EVs from patients with COVID-19 of varying severities and combined it with single-cell analysis of peripheral blood mononuclear cells. The authors identified a group of macrophage-related proteins enriched in refractory cases. Notably, they found that MACROH2A1, which was induced in monocytes and macrophages, is a biomarker for refractory COVID-19 pneumonia that is resistant to corticosteroids, emphasizing the value of integrating multi-omics approaches to understand complex disease mechanisms [63].

#### EVs in bacterial infections

EVs can mediate pathogen transmission during bacterial infections. For example, in *Legionella* infection, EVs from infected cells can spread to other cells and promote the expression of inflammatory cytokines [64]. In tuberculosis, antigen-presenting cells take up EVs containing mycobacterial antigens, thereby activating the acquired immune system [65–67]. EVs also directly activate the immune system and induce inflammation; in *Staphylococcus aureus* infections, neutrophil-derived EVs induce IL-6 and IL-1β production by macrophages [68]. In *Listeria* infections, EVs from infected dendritic cells induce stronger anti-pathogenic responses in recipient immature dendritic cells than those from uninfected cells [69]. These studies collectively highlight the pivotal role of EVs in bacterial infections, in not only facilitating pathogen transmission but also modulating immune responses and inflammation.

In the blood EV proteomics of bacterial infections (Table 1), Mehaffy et al. performed targeted proteomics (MRM-MS) on sera from 74 patients with latent tuberculosis infection (LTBI) and 29 controls, detecting *Mycobacterium tuberculosis*-derived peptides in 95% of patients with LTBI [70]. Another study compared the serum EV proteomes of six pediatric patients with pneumonia with those of healthy controls, revealing host response features such as neutrophil activation and complement regulation [71].

In summary, EVs play a significant role in immune response and tissue damage in infectious diseases by transporting either pathogens or derived proteins and inducing inflammation. Numerous molecules regulating pathological conditions have been identified through blood EV proteomics, making it one of the most active research areas outside oncology. Identifying biomarkers and therapeutic targets for clinical applications is highly anticipated.

## Conclusions and perspectives

The proteomics of blood EVs in inflammatory lung diseases can reveal valuable biomarkers, including key molecules related to disease pathogenesis, likely due to the functional characteristics of EVs and their advantages over serum or plasma as proteomic samples. Given the large volume of circulating blood passing through the lungs, organ-specific EVs may be more abundant in the bloodstream. EVs contain not only biomarkers but also crucial molecules that regulate pathological conditions; for instance, knockout mice lacking such molecules may display disease phenotypes [45]. Consequently, therapies targeting EV proteins can be developed using these proteins as companion biomarkers.

Several limitations of blood EV proteomics research persist. First, EV proteins are not readily captured in clinical practice using tools such as ELISA kits, and the identified biomarker molecules do not immediately translate into clinical applications. Developing assay systems for the direct detection of EV proteins is crucial, and progress has been made in this area [72, 73]. If trends in EV protein variations mirror those of whole serum or plasma proteins, these findings could be easily applied in clinical settings, as similar trends can be reproduced using ELISA on serum samples [74]. Second, the lack of standardized EV extraction protocols, immature proteomic analysis systems, and interpretation methods, along with developing techniques to improve the reproducibility of MS results, remain major challenges that must be addressed by future studies [6, 75–77]. To mitigate these issues, validating exploratory studies with additional cohorts is desirable.

In summary, blood EV proteomics for inflammatory respiratory diseases represents a promising research approach with potential applications in liquid biopsy and discovering novel therapies. The important findings discussed in this review highlight the significance of this approach. Further technological advances, such as the development of more reproducible proteomic assays and direct measurement systems for EVs, will contribute to progress in this field.

## Data Availability

Not applicable.

## Abbreviations

BA: Bronchial asthma
COPD: Chronic obstructive pulmonary disease
DDA: Data-dependent acquisition
DIA: Data-independent acquisition
EV: Extracellular vesicle
ILD: Interstitial lung disease
IPF: Idiopathic pulmonary fibrosis
LC–MS/MS: Liquid chromatography with tandem mass spectrometry
LTBI: Latent tuberculosis infection
MS: Mass spectrometry
PEA: Proximity extension assay
PF-ILD: Progressive-fibrosing interstitial lung diseases
PPF: Progressive pulmonary fibrosis
